# Strict biennial lifecycle and anthropogenic interventions affect temporal genetic differentiation in the endangered endemic plant, *Pedicularis hallaisanensis*

**DOI:** 10.3389/fpls.2024.1468395

**Published:** 2024-10-24

**Authors:** Seongjun Kim, Byoung-Doo Lee, Chang Woo Lee, Hwan-Joon Park, Jung Eun Hwang, Hyeong Bin Park, Young-Joong Kim, Daeyoung Jeon, Young-Jun Yoon

**Affiliations:** Research Center for Endangered Species, National Institute of Ecology, Yeongyang, Gyeongbuk, Republic of Korea

**Keywords:** anthropogenic intervention, biennial plant, genetic diversity, genotyping-by-sequencing, hemiparasitic herb, lousewort, single nucleotide polymorphism

## Abstract

Strict biennials are among the least known lifecycles in plant ecology due to their rarity in nature, and their population genetics still remain unknown. The present study addressed the strict biennial lifecycle and associated population genetics of *Pedicularis hallaisanensis*, an endangered endemic plant in Korea. All *P. hallaisanensis* individuals were counted in August from 2021 to 2023 in the wild population of Gayasan National Park, and lifecycle and morphological changes were monitored monthly. *A de novo* draft genome and single nucleotide polymorphism (SNP) analysis were used to study the population’s genetic structure. *P. hallaisanensis* strictly requires a 2-year lifecycle per generation, including 8 and 10 months of growing periods as a first-year seedling and second-year adult, respectively. Facultative annual and perennial lifecycles were undetected, resulting in odd-year and even-year flowering cohorts. Permutational multivariate analysis of variance on the detected 3,716 SNPs demonstrated that the flowering group (*p* < 0.005), microhabitat (*p* < 0.001), and their interaction (*p* < 0.01) had a significant effect on genetic structure, which was differentiated between odd-year and even-year flowering cohorts. Other cluster analyses also showed that a microhabitat under historical anthropogenic interventions contained lowered genetic diversity due to a decreased genetic distance between odd-year and even-year flowering cohorts (*p* < 0.05). Overall, the findings suggest that excessive anthropogenic interventions should be avoided to preserve genetic diversity in the wild *P. hallaisanensis* population. Moreover, conservation programs for similar biennial plants should collect wild breeds from both odd-year and even-year flowering cohorts to improve the genetic diversity of artificially propagated individuals.

## 1 Introduction

The lifecycle of endangered plants is important for conservation programs, as it reflects the legacy of adaptations to the surrounding environment (Niklas and Kutschera, 2010). The lifecycle of any plant species results from the optimization of resource allocation and utilization to enhance its fitness to the given habitat conditions (Kenrick, 2017). Since lifecycle strategy determines population dynamics and associated genetic diversity, understanding the lifecycle of endangered plants is critical for identifying potential bottlenecks in population growth and setting conservation priorities to sustain wild populations and habitats (Aronne, 2017; Tsuzuki et al., 2022). Although classifying lifecycles is primarily based on expected lifespan (annual, biennial, and perennial) and the total number of reproductive bouts throughout the lifespan (monocarpic and polycarpic), the diversity found in nature has caused many intermediate, idiosyncratic lifecycle patterns (e.g., facultative annual) of endangered plant species (Friedman, 2020).

Strict biennials are one of the least understood lifecycles in plant ecology because of their rarity under natural conditions (Friedman, 2020; Viswanathan and Aarssen, 2000). Several studies have described the lifecycle of biennial plants, such as *Pedicularis sylvatica* (Petrů, 2005) and *Gentianella campestris* (Glav Lundin and Eriksson, 2021). Such species strictly require a 2-year growing period prior to reproduction and death, in contrast to other facultative biennials, like winter annuals and monocarpic perennials, whose lifespan and reproduction depend on climatic event or growth rate (Bradshaw, 1978; Ter Bort et al., 1980; Kelly, 1985). This age dependence for reproduction also leads to the coexistence of two age classes (first-year seedling and second-year adult) within a single habitat (Kisdi, 2012), as well as the potential differentiation of cohorts flowering in either odd or even years (Petrů, 2005).

Recent advancements in genotyping-by-sequencing (GBS) have expanded knowledge regarding the population structure and genetic diversity of endangered plant species through fast, reliable processes at a low cost. This technique has helped identify genotype cohorts within a given endangered species according to single nucleotide polymorphism (SNP) loci (Cai et al., 2021; Wang et al., 2024). Nonetheless, GBS approaches for endangered strict biennial plants have principally focused on genetic variations across multiple habitats and population sizes, without considering their unique lifecycle patterns (Cho and Choi, 2011; Reisch and Hoiß, 2019). Since the differentiation in reproduction time may decelerate gene flow between cohorts flowering in odd and even years (Petrů, 2005; Rusterholz et al., 2023), genetic comparisons between odd- and even-year flowering cohorts are necessary for totally understanding the genetic diversity and population dynamics of biennial plants.

The present study addressed the strict biennial lifecycle and associated population genetics of *Pedicularis hallaisanensis* (Orobanchaceae), an endangered endemic plant species in Korea. It was hypothesized that *P. hallaisanensis* cohorts flowering in odd-year would differ in genetic structure from those flowering in even-year because the isolated reproduction times may limit gene flow between the two cohorts. To test this hypothesis, a wild *P. hallaisanensis* population was periodically monitored to confirm that it followed a strict biennial lifecycle. Given the unavailability of a reference nuclear genome for the target species, we assembled a *de novo* draft genome of *P. hallaisanensis* to detect SNPs and compare the genetic structure between the different flowering years.

## 2 Methods

### 2.1 Target species

**Figure 1:**
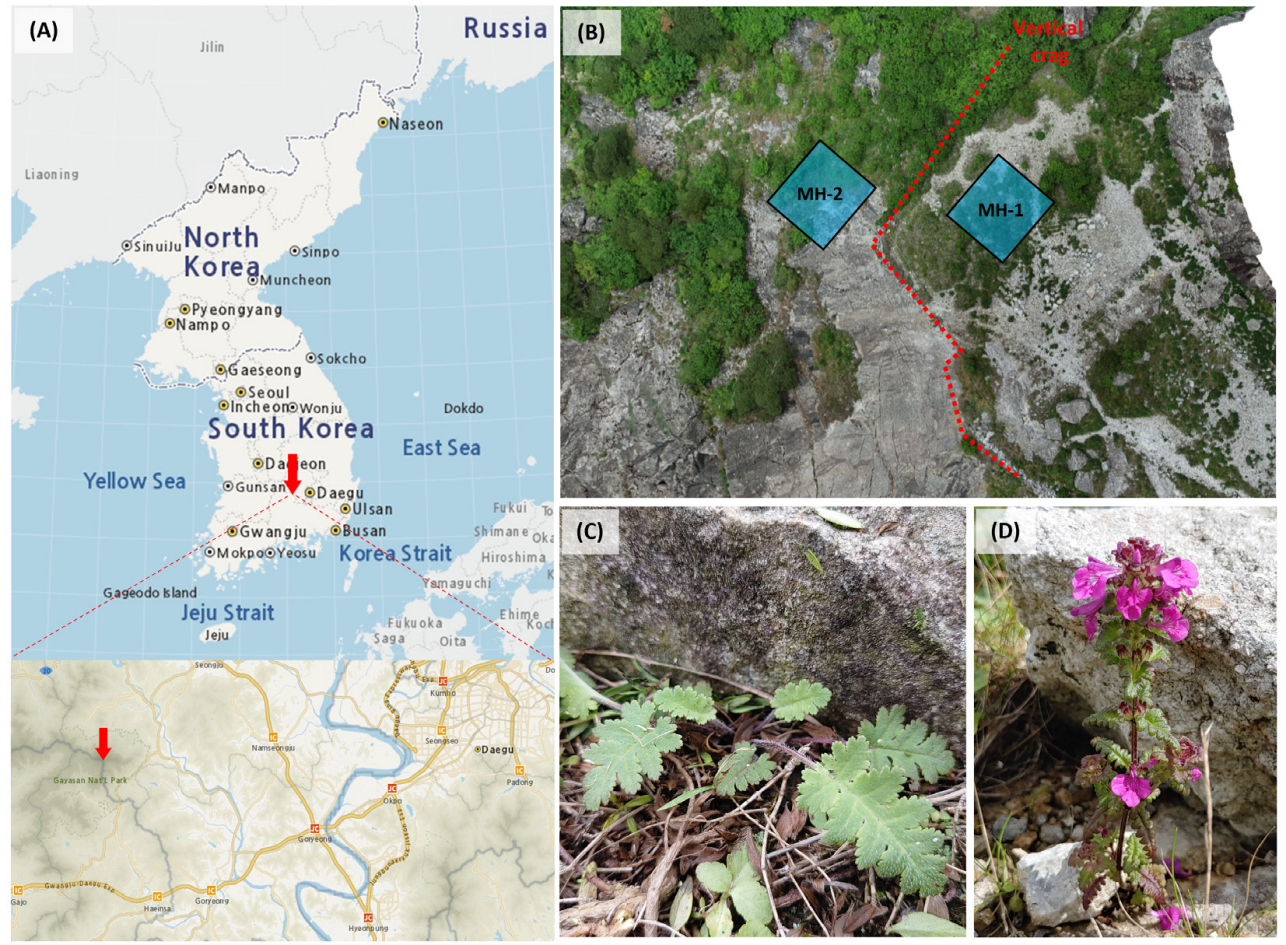
Location of the study area **(A)**, microhabitats (MH-1 and MH-2) for sampling of *Pedicularis hallaisanensis* SNP analysis in the study area **(B)**, and examples of first-year seedling **(C)** and a second-year adult **(D)** of *P. hallaisanensis*. (Source of **(A)**: https://map.ngii.go.kr/ms/map/NlipMap.do, accessed on 8 April 2024).

The target species is the root-hemiparasitic herb species, *P. hallaisanensis* (**Figures 1C, D**). This species is endemic to Korea and is legally protected as an endangered species (grade II) of the Ministry of Environment (Chung et al., 2023). It originally inhabited Korean mountaintop grasslands (altitude: 1,400–1,500 m) such as Hallasan and Gayasan (Kim et al., 2018), with voucher specimens stored at the herbariums of Inha University (Cho. 98454) and the Korea National Park Research Institute (Gaya_20160439), respectively (Cho and Choi, 2011; Han et al., 2022). However, the most wild populations have disappeared due to habitat loss and climate change (Kim et al., 2018). This species is known to be phylogenetically close to other hemiparasitic Orobanchaceae species, such as *Pedicularis spicata*, *Pedicularis verticillata, Pedicularis alaschanica*, and *Pedicularis longiflora*, according to previous ribosomal and chloroplast DNA studies (Cho and Choi, 2011; Cho et al., 2018; Zhang et al., 2020). Morphologically, *P. hallaisanensis* is distinguishable by its dense glandular hairs covering the entire body, a shorter galea compared to the lower lip, and three unequal calyx lobes when compared to other allied Orobanchaceae species (Cho and Choi, 2011; Kim et al., 2018). Although *P. hallaisanensis* has traditionally been speculated to be either an annual, biennial, or perennial species (Cho et al., 2018; Kim et al., 2018), our monitoring was the first to confirm that this species features a strict biennial lifecycle (see our Results section).

### 2.2 Study area and lifecycle monitoring

The study area was located in a mountaintop grassland within Gayasan National Park in South Korea (35°49′25″N, 128°7′10″E) (**Figures 1A, B**). The altitude of the study area ranges from 1,410 to 1,430 m above sea level (asl), with slopes varying between 0° and 20°. The average annual precipitation is 1,296 mm, with a relative humidity of 74.8% and an average air temperature of 7.6°C. The soil is less than 20 cm, lacking distinct evidence of horizon development (entisols), and is underlain by an impermeable bedrock layer. This was the only study area available for studying the target species, although we have investigated all known natural habitats around Hallasan, Gayasan, Seoraksan, and Bangtaesan from 2019 to 2023 in search of additional wild *P. hallaisanensis* populations. National inventory data from the National Institute of Ecology have also recorded no wild *P. hallaisanensis* population remaining in any natural habitats since 2020, except for this study area.

All *P. hallaisanensis* individuals were counted in August from 2021 to 2023 to quantify the population size in the study area. Of these, one individual was used for the draft genome analysis, and 20 were used for SNP analyses. All *P. hallaisanensis* individuals in the study area were sorted into age classes according to the elapsed time after germination (first-year seedling and second-year adult). Each *P. hallaisanensis* was labeled and monthly monitored to track any morphological changes throughout the biennial lifecycle during the growing season (April–November).

### 2.3 DNA extraction and draft genome assembly

Fresh leaves of a *P. hallaisanensis* individual were sampled in June 2023, stored in an icebox (4°C), and brought to the laboratory for draft genome assembly. Genomic DNA was extracted from the leaf samples using the Aprep Total DNA KIT (APBIO, Namyangju, South Korea) based on the manufacturer’s instructions. The extracted DNA was then quantified with a Thermo Scientific Nanodrop 8000 spectrophotometer (Fisher Scientific, Waltham, MA, USA), digested using the ApeKI enzyme (GCWGC), and reorganized into short reads of 151 bp length, following the protocols of Elshire et al. (2011) and Oh et al. (2023). Sequencing was conducted using the Illumina Hiseq X Ten Platform (Illumina Inc., San Diego, CA, USA). Illumina raw reads were filtered using Trimmomatic v.0.39 to exclude poor-quality reads (window size: 4, mean quality: ≧ 15, leading and trailing: ≧ 3, read length: ≧ 36 bp) (Bolger et al., 2014), and *de novo* assembly was done using SOAPdenovo2 v.2.04 (K-mer = 69) (Luo et al., 2012).

### 2.4 SNP detection and filtration

Two 5 × 5 m plots were established for SNP analyses in two different microhabitats (MH-1 and MH-2). Although the distance between these two microhabitats was only 20 m, they differed in terms of historical anthropogenic interventions (**Figure 1B**). MH-1 had been artificially flattened and managed as a heliport until the early 2010s (**Supplementary Figure S1**), but it has recently become revegetated after such management was stopped. Conversely, MH-2 was located outside the old heliport sites and was relatively sheltered from heavy anthropogenic interventions. Both MH-1 and MH-2 contained both odd-year-flowering (OYF) and even-year-flowering (EYF) *P. hallaisanensis* cohorts, in contrast to several other microhabitats, which included either OYF or EYF cohorts only.

Fresh cauline leaves from five OYF and five EYF were randomly sampled from each plot for SNP detection in June 2023 and 2022, respectively (*n* = 20). Short DNA reads (151 bp) were obtained using the protocols of Elshire et al. (2011) and Oh et al. (2023) and sequenced with the Illumina Hiseq X Ten platform (Illumina Inc., CA, USA). Low-quality raw reads were then removed using cutadapt v.1.8.3 (Li, 2013) and Trimmomatic v.0.39 (Bolger et al., 2014). Filtered clean reads were mapped to the assembled draft genome of *P. hallaisanensis* using BWA v.0.7.17-r1188 (Li, 2013), and raw SNPs were detected using SAMtools v.0.1.16 (Li et al., 2009). To ensure SNP quality, only biallelic SNP loci without any missing values throughout all 20 samples were selected for further statistical analyses (3716 SNPs in total).

### 2.5 Statistical analyses

To describe the genetic diversity, Nei’s genetic diversity (GD), polymorphism informative content (PIC), minor allele frequency (MAF), and observed heterozygosity (Ho) were calculated using the snpReady package in R v.4.3.2 software (Granto et al., 2018).

Permutational multivariate analysis of variance (PERMANOVA) and permutational analysis of multivariate dispersion (PERMDISP) were conducted using Bray–Curtis dissimilarity based on 9,999 permutations to test the effects of flowering group (OYF or EYF) and microhabitat (MH-1 or MH-2) on the multivariate genetic centroids and dispersions of the SNP data from 20 P*. hallaisanensis* samples (*α* = 0.05). Nonmetric multidimensional ordination scaling (NMDS) was further conducted using Bray–Curtis dissimilarity to visualize the multivariate genetic variability, and a general linear model (GLM) was applied to test the relationship between NMDS axes, flowering group, and microhabitat (*n* = 20, *α* = 0.05). These analyses were performed using the vegan (Oksanen et al., 2024) and agricolae (De Mendiburu and Simon, 2015) packages in R v.4.3.2 software (R Core Team, 2023).

Neighbor-joining method and k-means clustering of the silhouette width approach were implemented using the factoextra (Kassambara and Mundt, 2016) and ape (Paradis et al., 2004) packages in R v.4.3.2 software to show genetic differences among the sampled *P. hallaisanensis* (*n* = 20) (Batool and Henning, 2021). STRUCTURE 2.3.4 software was also used with 10,000 burn-in periods, 100,000 Markov chain Monte Carlo (MCMC) replications, and 10 iterations to estimate the membership probability of each *P. hallaisanensis* sample according to hypothetical ancestral genotypes (Pritchard et al., 2000).

## 3 Results

### 3.1 Lifecycle and population size of *P. hallaisanensis*

**Figure 2:**
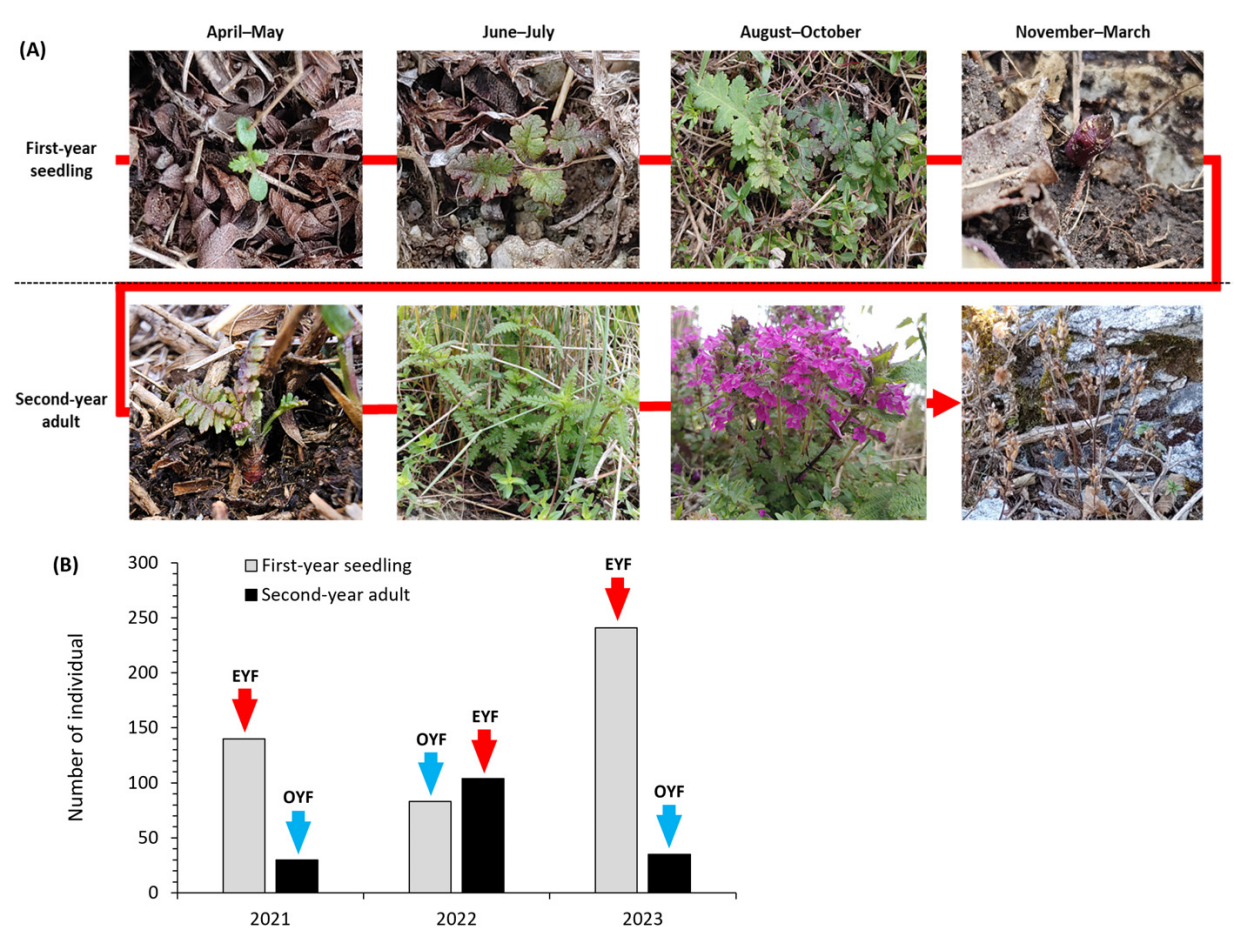
**(A)** Summary of the strict biennial lifecycle of *Pedicularis hallaisanensis* and **(B)** number of first-year seedling and second-year adult of *P. hallaisanensis* individuals in the study area. Arrows above the bars in **(B)** indicate odd-year-flowering (OYF) and even-year-flowering (EYF) *P. hallaisanensis*.

Our monitoring demonstrated that *P. hallaisanensis* strictly required 2 years of lifecycle per generation, with approximately 8 and 10 months of growing periods as first-year seedling and second-year adult, respectively. From April to May, first-year seedlings of *P. hallaisanensis* germinated and developed leaves and roots, eventually forming overwintering buds in November (**Figure 2A**). There were only rosette leaves without distinguishable shoots and cauline leaves in the aboveground of the first-year seedlings (**Figure 2A**). However, second-year adults of *P. hallaisanensis* rapidly established shoots and cauline leaves from the overwintering buds starting in April. Flowering and seed production of occurred from August to November in the second year, and all second-year adults died after producing seeds (**Figure 2A**). Facultative annual and perennial lifecycle patterns were undetected for *P. hallaisanensis* in the study area. Accordingly, a flowering event of *P. hallaisanensis* occurred biennially at several microhabitats (other than MH-1 and MH-2), when they contained only first-year seedlings or second-year adults.

The total number of *P. hallaisanensis* individuals in the study area was 170, 187, and 276 in 2021, 2022, and 2023, respectively (**Figure 2B**). First-year seedlings and second-year adults accounted for 44.4%–87.3% and 12.7%–55.6% of the *P. hallaisanensis* population, respectively. EYF consistently showed a larger number of *P. hallaisanensis* individuals than OYF regardless of age throughout the monitoring period (**Figure 2B**).

### 3.2 Draft genome and filtered SNPs

Sequencing of *P. hallaisanensis* provided 100.9 Gb of Illumina short reads, which were used to assemble the reference draft genome, involving 2.6 million contigs with a total length of 1.23 Gb and an N50 length of 0.54 Mb, for further SNP identification (**Supplementary Table S1**). Subsequently, a total of 123.7 Tb of Illumina short reads was obtained from 10 OYF and 10 EYF samples (20 genotypes), from which 3,716 filtered SNPs were identified to analyze the genetic structure across flowering groups and microhabitats. Genetic diversity indices for 3,716 SNPs are described in **Supplementary Table S2.**

### 3.3 Effects of flowering group and microhabitat

**Figure 3:**
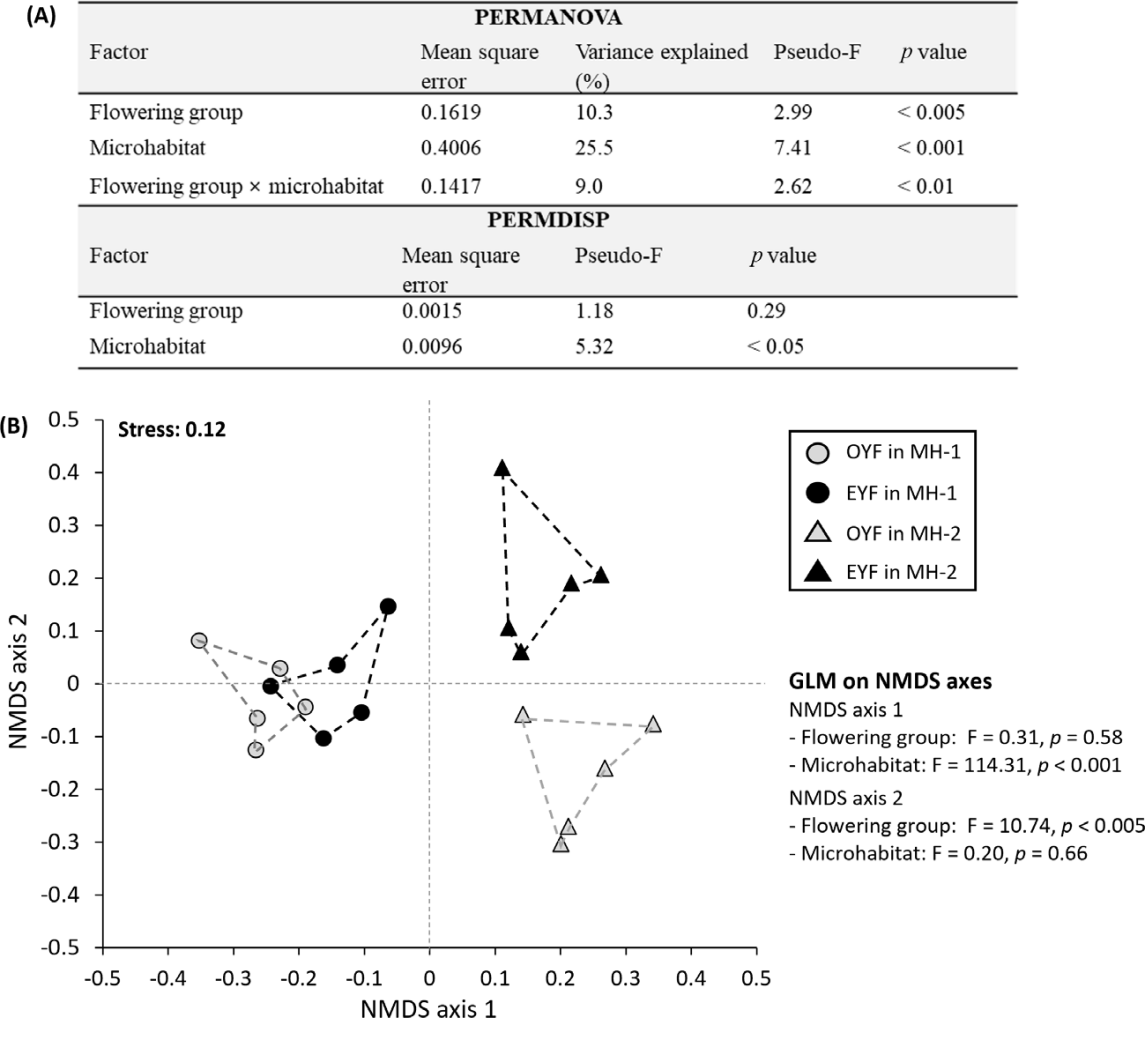
**(A)** Results of permutational multivariate analysis of variance (PERMANOVA) and permutational analysis of multivariate dispersion (PERMDISP). **(B)** Nonmetric multidimensional ordination scaling (NMDS; *n* = 20) and general linear model (GLM; *n* = 20) on each NMDS axis using 3,716 SNP data from odd-year-flowering (OYF) and even-year-flowering (EYF) *Pedicularis hallaisanensis* in the two studied microhabitats (MH-1 and MH-2).

PERMANOVA on the 3,716 SNPs demonstrated that the flowering group (*p* < 0.005), microhabitat (*p* < 0.001), and their interaction (*p* < 0.01) had a significant effect on the multivariate centroid of 3,716 SNPs from *P. hallaisanensis* (**Figure 3A**). These three factors explained 44.8% of the multivariate variances in SNPs, while the remaining 55.2% of the variance were attributed to within-group variabilities. On the other hand, PERMDISP indicated that only microhabitat (*p* < 0.05) had a significant effect on the multivariate dispersion of 3,716 SNPs from *P. hallaisanensis* (**Figure 3A**).

NMDS ordination showed a similar pattern with PERMANOVA and PERMDISP (stress value: 0.12, **Figure 3B**). Additional GLM on NMDS axes found that axes 1 and 2 represented the variabilities resulting from microhabitat (*p* < 0.001) and flowering group (*p* < 0.005), respectively (**Figure 3B**). Since the multivariate centroids of OYF and EYF were closer in MH-1 than in MH-2 (**Figure 3B**), the multivariate dispersion conversely became larger in MH-2 regardless of the similar genetic diversity indices among the four combinations of OYF and EYF in MH-1 and MH-2 (**Supplementary Table S2**).

### 3.4 Genetic clustering

**Figure 4:**
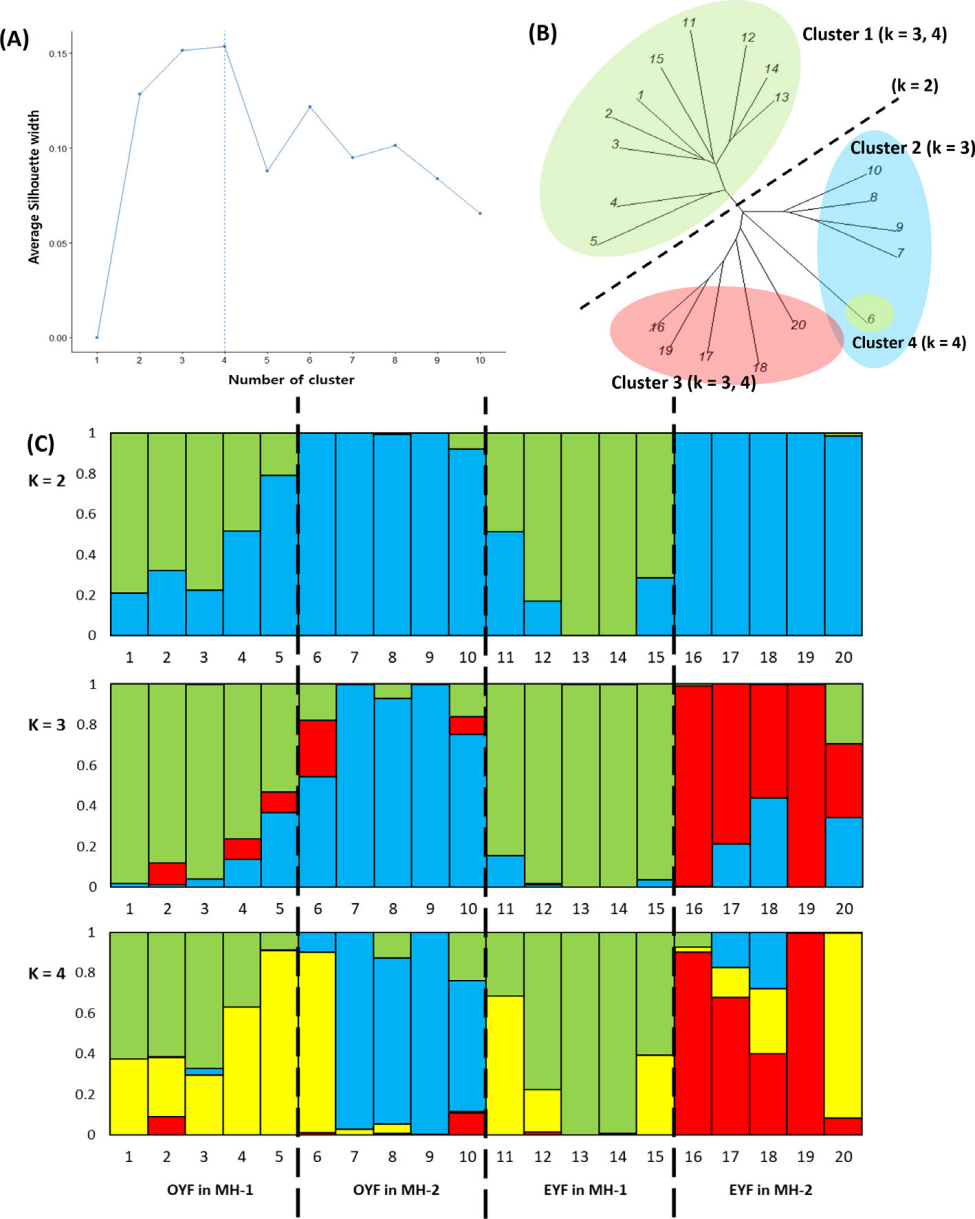
**(A)** Selection of the number of clusters using the silhouette width criterion (k), **(B)** neighbor-joining phylogenetic tree, and **(C)** STRUCTURE analysis results on 3,716 SNP data from odd-year-flowering (OYF; MH-1: 1–5, MH-2: 6–10) and even-year-flowering (EYF; MH-1: 11–15, MH-2: 16–20) *Pedicularis hallaisanensis* **(B)** in the two studied microhabitats (MH-1 and MH-2). The colors of the bar graphs in **(C)** imply the different clusters estimated by STRUCTURE analysis.

The silhouette width approach suggested that the optimum K number was four, followed by three and two (**Figure 4A**). Neighbor-joining phylogenetic tree and k-means clustering revealed that the 20 genotypes of *P. hallaisanensis* were divided by microhabitat (MH-1 and MH-2) at *K* = 2. At *K* = 3, the genotypes in MH-2 were subdivided by flowering group (OYF and EYF) (**Figure 4B**). Only one of the genotypes of OYF in MH-2 was separated as an additional cluster at *K* = 4, while the subdivisions between OYF and EYF in MH-1 remained relatively unclear (**Figure 4B**). The STRUCTURE analysis exhibited a similar pattern with the neighbor-joining phylogenetic tree, including the differentiation between MH-1 and MH-2 at *K* = 2 (**Figure 4C**), and a clearer subdivision by the flowering group in MH-2 than in MH-1 at *K* = 3 and 4 (**Figure 4C**).

## 4 Discussion

Strict biennial plant species have a distinctive lifecycle compared to annual and facultative biennial plants, characterized by an extended time period for each generation (2 years from seed germination to flowering and death) (Kelly, 1985). All the studied *P. hallaisanensis* required a 2-year period from seed germination to blooming, seed production, and death (**Figure 2A**), which fits into the biennial lifecycle patterns of other *Pedicularis* species in alpine meadows (Petrů, 2005; Zhang et al., 2011). Our results support several expectations on biennial lifecycle in nature. For example, flowers of *P. hallaisanensis* were detected every year within the study area (**Figure 2B**), confirming that strict biennial plants can establish two distinct cohorts with different flowering periods (odd or even years), sharing the same microhabitat (Kelly, 1985; Petrů, 2005). Our results are in line with the mathematical studies (Davydova et al., 2003; Kisdi, 2012), which anticipate the coexistence of first-year seedlings and second-year adults of biennial plants within a given time frame and microhabitat. The detected annual oscillations in the number of flowering second-year adults are also consistent with previous findings on other strict biennial plants by Anderson et al. (2021) and Petrů (2005) (**Figure 2B**).

Temporal genetic differentiation in plant ecology is traditionally considered to be the evolutionary changes in population genetics over long periods or the short-term genetic constitutions caused by natural and anthropogenic disturbances along several generations (Linhart and Grant, 1996; Stadler et al., 2010; Gómez et al., 2018). It may also reflect the genetic variability within a perennial plant population due to the complexity of its age structure and intraseasonal variations in flowering phenology (Reed et al., 2022; Tsuzuki et al., 2022). In the present study, multivariate comparisons using the detected SNPs showed that the flowering group had a significant effect on the genetic structure of *P. hallaisanensis* (**Figure 3**). Due to the absence of comparable studies on the population genetics of strict biennial plants, it is uncertain whether the observed genetic differentiation is consistent across plant species with similar lifecycle characteristics (Petrů, 2005; Zhang et al., 2011; Anderson et al., 2021; Glav Lundin and Eriksson, 2021). Therefore, further studies should address this uncertainty in other biennial *Pedicularis* species to totally confirm if the detected temporal genetic differentiation can be generalized. Nonetheless, the findings enable us to expect that the strict biennial lifecycle may contribute to temporal genetic differentiation by creating two different cohorts that flower in either odd or even years within a single habitat, in contrast to the interannual maintenance of genetic structure observed in facultative biennial plant species (Valverde et al., 2016).

There was a significant effect of microhabitat, which was even greater than that of the flowering group (**Figure 3A**). The magnitude of temporal genetic differentiation due to the strict biennial lifecycle also depended on the spatial location of the individuals, as evidenced by the closer genetic distance between OYF and EYF individuals within the same microhabitat, compared to that between OYFs (or EYFs) in different microhabitats (**Figures 3B**, **4B**). These patterns indicate that gene flow between the flowering groups is likely to occur, at least within a single microhabitat (Rusterholz et al., 2023). The detailed mechanisms behind these patterns are currently unknown. However, eventual seed dispersal and dormancy may allow the gene flow between OYF and EYF, potentially confounding the observed temporal genetic differentiation (Valverde et al., 2016; Glav Lundin and Eriksson, 2021). This is despite the fact that most seeds of biennial *Pedicularis* plants are known to germinate in the first spring after they are produced (Kelly, 1985; Petrů, 2005; Kim et al., 2019). The significant difference between microhabitats suggests that gene flow between MH-1 and MH-2 may have been limited, despite the small habitat area, allowing small-scale spatiotemporal changes in genetic structure to become detectable (Chan et al., 2018; Zhang et al., 2021).

It is also notable that flowering group × microhabitat interaction played a marked role in the genetic diversity across the studied *P. hallaisanensis* cohorts (**Figure 3A**). The genetic variability between OYF and EYF was remarkable in MH-2, in contrast to the relatively unclear genetic differences in MH-1 (**Figures 3B**, **4**). These patterns further induced the lowered multivariate dispersions in MH-1 (**Figure 3**), reflecting the reduction of genetic diversity in MH-1 (Anderson et al., 2006). These patterns might be related to the previous anthropogenic interventions around the heliport near MH-1 (**Supplementary Figure S1**), given that extreme disturbance events can reduce the genetic diversity of short-lived herb populations by increasing the self-compatible reproduction rate and confounding the species’ lifecycle pattern (Quintana-Asencio et al., 2011; González et al., 2019; Almeida-Rocha et al., 2020; Anderson et al., 2021). Given the hemiparasitic characteristics of *P. hallaisanensis*, historical disturbances to the surrounding host plants may have also impacted the health and genetic diversity of *P. hallaisanensis*, particularly in MH-1 (Kim et al., 2019). Therefore, the findings allow us to suspect that excessive anthropogenic interventions could reduce the genetic diversity of strict biennial plant populations by hindering genetic differentiation between OYF and EYF cohorts. However, moderate disturbances, such as grazing and mowing, may promote seedling recruitment in biennial *Pedicularis* species by helping to maintain grassland habitats (Petrů, 2005).

In summary, this study is the first to record the strict biennial lifecycle and associated genetic variabilities of the endangered endemic plant species, *P. hallaisanensis*, using GBS and SNPs. Our results showed temporal genetic differentiation between OYF and EYF, which may contribute to the genetic diversity of the remaining *P. hallaisanensis* population. Such genetic differentiation, driven by the biennial lifecycle, varied across microhabitats and may be related to differing levels of historical anthropogenic interventions. In this context, excessively strong anthropogenic interventions in the *P. hallaisanensis* habitat should be avoided to preserve the genetic diversity of the wild population. Instead, moderate management practices like mowing and grazing can help protect mountain grassland habitats from the invasion of competitive shrubs and trees (Petrů, 2005; Kim et al., 2018). Moreover, future conservation programs should collect original wild breeds from both OYF and EYF to ensure that genetic diversity in the wild population is passed on to artificially propagated *P. hallaisanensis*. The detected biennial lifecycle is also remarkable for *ex situ* conservation, highlighting the necessity of 2-year cultivation cycles to successfully propagate collected *P. hallaisanensis* (Kim et al., 2019).

## Data Availability

The datasets presented in this study can be found in online repositories. The names of the repository/repositories and accession number(s) can be found below: https://www.ncbi.nlm.nih.gov/, PRJNA1136402.

## References

[B1] Almeida-Rocha J. M. Soares L. A. S. S. Andrade E. R. Gaiotto F. A. Cazetta E. (2020). The impact of anthropogenic disturbances on the genetic diversity of terrestrial species: A global meta-analysis. Mol. Ecol. 29, 4812–4822. doi: 10.1111/mec.15688 33058295

[B2] Anderson M. Ellingsen K. E. AcArdle B. H. (2006). Multivariate dispersion as a measure of beta diversity. Ecol. Lett. 9, 683–693. doi: 10.1111/j.1461-0248.2006.00926.x 16706913

[B3] Anderson R. C. Anderson M. R. Bauer J. T. Loebach C. Mullarkey A. Engelhardt M. (2021). Response of the invasive *Alliaria petiolata* to extreme temperatures and drought. Ecosphere 12, e03510. doi: 10.1002/ecs2.3510

[B4] Aronne G. (2017). Identification of bottlenecks in the plant life cycle for sustainable conservation of rare and endangered species. Front. Ecol. Evol. 5, 76. doi: 10.3389/fevo.2017.00076

[B5] Batool F. Henning C. (2021). Clustering with the average Silhouette width. Comput. Stat. Data Anal. 158, 107190. doi: 10.1016/j.csda.2021.107190

[B6] Bolger A. M. Lohse M. Usadel B. (2014). Trimmomatic: A flexible trimmer for Illumina sequence data. Bioinformatics 30, 2114–2120. doi: 10.1093/bioinformatics/btu170 24695404 PMC4103590

[B7] Bradshaw M. D. (1978). Plant population studies and their relevance to nature conservation. Biol. Conserv. 14, 223–242. doi: 10.1016/0006-3207(78)90012-5

[B8] Cai C. Xiao J. Ci X. Conran J. G. Li J. (2021). Genetic diversity of *Horsfieldia tetratepala* (Myristicaceae), an endangered plant species with extremely small populations to China: Implications for its conservation. Plant Syst. Evol. 307, 50. doi: 10.1007/s00606-021-01774-z

[B9] Chan Y. M. Tnah L. H. Lee S. L. Bhassu S. Lee C. T. Chua L. W. L. (2018). Limited dispersal and geographic barriers cause population differentiation and structuring in *Begonia maxwelliana* at both large and small scales. Plant Ecol. Divers. 11, 69–83. doi: 10.1080/17550874.2018.1471625

[B10] Cho W.-B. Choi B.-H. (2011). Taxonomic position of *Pedicularis hallaisanensis* Hurusawa, an endemic plant of Mt. Halla. Korean J. Plant Taxon. 41, 130–137. doi: 10.11110/kjpt.2011.41.2.130

[B11] Cho W.-B. Lee D.-H. Choi I.-S. Lee J.-H. (2018). The complete chloroplast genome of hemi-parasitic *Pedicularis hallaisanensis* (Orobanchaceae). Mitochondrial DNA Part B: Resour. 3, 235–236. doi: 10.1080/23802359.2018.1437820 PMC7800071 33474128

[B12] Chung G. Y. Jang H. D. Chang K. S. Choi H. J. Kim Y.-S. Kim H.-J.. (2023). A checklist of endemic plants on the Korean Peninsula II. Korean J. Plant Taxon. 53, 79–101. doi: 10.11110/kjpt.2023.53.2.79

[B13] Davydova N. V. Diekmann O. van Gils S. A. (2003). Year class coexistence or competitive exclusion for strict biennials? J. Math. Biol. 46, 95–131. doi: 10.1007/s00285-002-0167-5 12567230

[B14] De Mendiburu F. Simon R. (2015). Agricolae - ten years of an open source statistical tool for experiments in breeding, agriculture and biology. PeerJ Preprints. Peerj.preprints. 1404v1. doi: 10.7287/peerj.preprints.1404v1

[B15] Elshire R. J. Glaubitz J. C. Sun Q. Poland J. A. Kawamoto K. Buckler E. S.. (2011). A robust, simple gynotyping-by-sequencing (GBS) approach for high diversity species. PloS One 6, e19379. doi: 10.1371/journal.pone.0019379 21573248 PMC3087801

[B16] Friedman J. (2020). The evolution of annual and perennial plant life histories: Ecological correlates and genetic mechanisms. Annu. Rev. Ecol. Evol. Syst. 51, 461–481. doi: 10.1146/annurev-ecolsys-110218-024638

[B17] Glav Lundin L. Eriksson O. (2021). The decline of *Gentianella campestris*: Three decades of population development of an endangered grassland plant in Sweden. Nord. J. Bot. 39, e03007. doi: 10.1111/njb.03007

[B18] Gómez R. Méndez-Vigo B. Marcer A. Alonso-Blanco C. Picó F. X. (2018). Quantifying temporal change in plant population attributes: Insights from a resurrection approach. AOB Plants 10, ply063. doi: 10.1093/aobpla/ply063 30370042 PMC6198925

[B19] González A. V. Gómez-Silva V. Ramírez M. J. Fontúrbel F. E. (2019). Meta-analysis of the differential effects of habitat fragmentation and degradation on plant genetic diversity. Conserv. Biol. 34, 711–720. doi: 10.1111/cobi.13422 31605401

[B20] Granto I. S. C. Galli G. De Oliveira Couto E. G. E Souza M. B. Mendonca L. F. Fritsche-Neto R. (2018). snpReady: A tool to assist breeders in genomic analysis. Mol. Breed. 38, 102. doi: 10.1007/s11032-018-0844-8

[B21] Han S. Leem H. Jang H.-D. Kim Y.-Y. So S. (2022). Floristic study of Gayasan National Park in Korea. Korean J. Plant Res. 35, 248–288. doi: 10.7732/kjpr.2022.35.2.248

[B22] Kassambara A. Mundt F. (2016). Package ‘factoextra.’ Package, version 1.0.3.

[B23] Kelly D. (1985). On strict and facultative biennials. Oecologia 67, 292–294. doi: 10.1007/BF00384302 28311327

[B24] Kenrick P. (2017). Changing expressions: A hypothesis for the origin of the vascular plant life cycle. Phil. Trans. R. Soc B 373, 20170149. doi: 10.1098/rstb.2017.0149 PMC5745341 29254970

[B25] Kim L.-K. Choi S.-D. Choo G.-C. Hwang B.-Y. Gang G.-H. So S.-K.. (2018). Environmental characteristics and floristic study of endangered *Pedicularis hallaisanensis* habitats. J. Agricul. Life Sci. 52, 163–173. doi: 10.14397/jals.

[B26] Kim L.-K. Park E.-H. Gang G. Hwang B.-Y. Jung H.-J. Kim M.-Y.. (2019). Form and embryonic characteristics of *Pedicularis hallaisanensis* seeds as endangered wild species II-class using host plant. J. Korean Soc For. Sci. 108, 290–295. doi: 10.14578/jkfs.2019.108.3.290

[B27] Kisdi É. (2012). Year-class coexistence in biennial plants. Theor. Popul. Biol. 82, 18–21. doi: 10.1016/j.tpb.2012.03.003 22838024

[B28] Li H. (2013). Aligning sequence reads, clone sequences and assembly contigs with BWA-MEM. Priprint at arXiv. arXiv 1303, 3997v2. doi: 10.48550/arXiv.1303.3997

[B29] Li H. Handsaker B. Wysoker A. Fennell T. Ruan J. Homer N.. (2009). The sequence alignment/map format and SAMtools. Bioinformatics 25, 2078–2079. doi: 10.1093/bioinformatics/btp352 19505943 PMC2723002

[B30] Linhart Y. B. Grant M. C. (1996). Evolutionary significance of local genetic differentiation in plants. Annu. Rev. Syst. 27, 237–277. doi: 10.1146/annurev.ecolsys.27.1.237

[B31] Luo R. Liu B. Xie Y. Li Z. Huang W. Yuan J.. (2012). SOAPdenovo2: An empirically improved memory-efficient short-read *de novo* assembler. GigaScience 1, 18. doi: 10.1186/2047-217X-1-18 23587118 PMC3626529

[B32] Niklas K. J. Kutschera U. (2010). The evolution of the land plant life cycle. New Phytol. 185, 27–41. doi: 10.1111/j.1469-8137.2009.03054.x 19863728

[B33] Oh J.-E. Kim J.-E. Kim J. Lee M.-H. Lee K. Kim T.-H.. (2023). Development of an SNP marker set for marker-assisted backcrossing using genotyping-by-sequencing in tetraploid perilla. Mol. Genet. Genomics 298, 1435–1447. doi: 10.1007/s00438-023-02066-6 37725237

[B34] Oksanen J. Simpson G. L. Blanchet F. G. Kindt R. Legendre P. Minchin P. R.. (2024). Package ‘vegan.’ Community ecology package, version 2.6-6.

[B35] Paradis E. Claude J. Strimmer K. (2004). APE: Analysis of phylogenetics and evolution in R language. Bioinformatics 20, 289–290. doi: 10.1093/bioinformatics/btg412 14734327

[B36] Petrů M. (2005). Year-to-year oscillations in demography of the strictly biennial *Pedicularis sylvatica* and effects of experimental disturbance. Plant Ecol. 181, 289–298. doi: 10.1007/s11258-005-7223-3

[B37] Pritchard J. K. Stephens M. Donnelly P. (2000). Inference of population structure using multilocus genotype data. Genetics 155, 945–959. doi: 10.1093/genetics/155.2.945 10835412 PMC1461096

[B38] Quintana-Asencio P. F. Menges E. S. Weekley C. W. Kelrick M. I. Pace-Aldana B. (2011). Biennial cycling caused by demographic delays in a fire-adapted annual plant. Popul. Ecol. 53, 131–142. doi: 10.1007/s10144-010-0228-3

[B39] R Core Team. (2023). R: A language and environment for statistical computing. Vienna, Austria: R Foundation for Statistical Computing. Available online at: https://www.R-project.org/.

[B40] Reed W. J. Ison J. L. Waananen A. Shaw F. H. Wagenius S. Shaw R. G. (2022). Genetic variation in reproductive timing in a long-lived herbaceous perennial. Am. J. Bot. 109, 1861–1874. doi: 10.1002/ajb2.16072 36112607

[B41] Reisch C. Hoiß B. (2019). Genetic variation of Gentianella campestris ssp. *Campestris* in the Northern Alps: How important are population size and isolation. Alp. Bot. 129, 11–20. doi: 10.1007/s00035-019-00216-4

[B42] Rusterholz H.-P. Ursenbacher S. Weibel U. Coray A. Baur B. (2023). Genetic diversity and population structure derived from body remains of the endangered flightless longhorn beetle *Iberodorcadion fuliginator* in grassland fragments in Central Europe. Diversity 15, 16. doi: 10.3390/d15010016

[B43] Stadler K. Koch M. Bernherdt K.-G. Greimler J. (2010). Spatial arrangement and genetic structure in *Gentianella aspera* in a regional, local, and temporal context. Plant Syst. Evol. 286, 7–19. doi: 10.1007/s00606-010-0274-5

[B44] Ter Bort S. J. Janse A. Kwak M. M. (1980). Life cycle variation in *Pedicularis palustris* L. (Scrophulariaceae). Acta Bot. Neerl. 29, 397–405. doi: 10.1111/plb.1980.29.issue-5-6

[B45] Tsuzuki Y. Takada T. Ohara M. (2022). Modeling temporal dynamics of genetic diversity in stage-structured plant populations with reference to demographic genetic structure. Theor. Popul. Biol. 148, 76–85. doi: 10.1016/j.tpb.2022.11.001 36402453

[B46] Valverde J. Gómez J. M. García C. Sharbel T. F. Jiménez M. N. Perectti F. (2016). Inter-annual maintenance of the fine-scale genetic structure in a biennial plant. Sci. Rep. 6, 37712. doi: 10.1038/srep37712 27883087 PMC5121606

[B47] Viswanathan D. Aarssen L. W. (2000). Why biennials are so few: Habitat availability and the species pool. Ecoscience 7, 461–465. doi: 10.1080/11956860.2000.11682618

[B48] Wang Y. Li H. Yang Z. Liu B. Liu Y. Zhong Y. (2024). Genotyping-by-sequencing study of the genetic diversity and population structure of the endangered plant *Tsoongiodendron odorum* Chun in China. Forests 15, 910. doi: 10.3390/f15060910

[B49] Zhang L. Guo H. Wang M. Du G. (2011). Plasticity of reproductive traits responding to variation in light availability at the rosette stage of the first year in a strict biennial, *Pedicularis torta*, from a field on the Qinghai-Tibet Plateau, China. Plant Species Biol. 26, 105–110. doi: 10.1111/j.1442-1984.2010.00305.x

[B50] Zhang R. Xu B. Li J. Zhao Z. Han J. Lei Y.. (2020). Transit from autotrophism to heterotrophism: Sequence variation and evolution of chloroplast genomes in Orobanchaceae species. Front. Genet. 11, 542017. doi: 10.3389/fgene.2020.542017 33133143 PMC7573133

[B51] Zhang X. Yang L. Liu Y.-H. Zhou X.-L. Zhang L.-Q. Wang Y.-H.. (2021). Genetic diversity, genetic structure, and demographic history of *Cinnamomum chago*, a plant species with extremely small populations in China. Glob. Ecol. Conserv. 31, e01808. doi: 10.1016/j.gecco.2021.e01808

