# Supplementary material for: Strict biennial lifecycle and anthropogenic interventions affect temporal genetic differentiation in the endangered endemic plant, Pedicularis hallaisanensis
Source: Front Plant Sci. 2024 Oct 24;15:1468395. doi: 10.3389/fpls.2024.1468395 (PMC11542022; doi:10.3389/fpls.2024.1468395)
Supplement: Supplementary file 1 [file DataSheet1.docx]

Supplementary Material

Table S1. Information of Illumina sequencing and de novo assembly to acquire *Pedicularis hallaisanensis* draft genome.

| Illumina sequencing | |
| --- | --- |
| K-mer | 69 |
| Number of read | 668,230,386 |
| Average read length | 151 |
| Total length of sequencing data (Gbp) | 100.90 |
| GC content (%) | 40.75 |
| Q30 (%) | 92.97 |
| Assembled draft genome | |
| Total number of contigs | 2,613,976 |
| Average contig size (bp) | 471 |
| Minimum contig size (bp) | 200 |
| Maximum contig size (bp) | 100,525 |
| Total length of draft genome (Gbp) | 1.23 |
| N50 (bp) | 545 |

Table S2. Diversity indices based on 3716 SNPs from odd-year-flowering (OYF) and even-year-flowering (EYF) *Pedicularis hallaisanensis* in the two microhabitats (MH-1 and MH-2).

| Flowering group | Microhabitat | n | GD | Ho | MAF | PIC |
| --- | --- | --- | --- | --- | --- | --- |
| OYF | MH-1 | 5 | 0.327 | 0.369 | 0.260 | 0.255 |
|  | MH-2 | 5 | 0.308 | 0.295 | 0.243 | 0.242 |
| EYF | MH-1 | 5 | 0.306 | 0.357 | 0.240 | 0.241 |
|  | MH-2 | 5 | 0.324 | 0.272 | 0.254 | 0.255 |
| Whole genotypes | | 20 | 0.405 | 0.323 | 0.321 | 0.316 |

n: number of genotypes (individuals); GD: Nei’s genetic diversity; Ho: observed heterozygosity; MAF: minor allele frequency; PIC: polymorphism informative


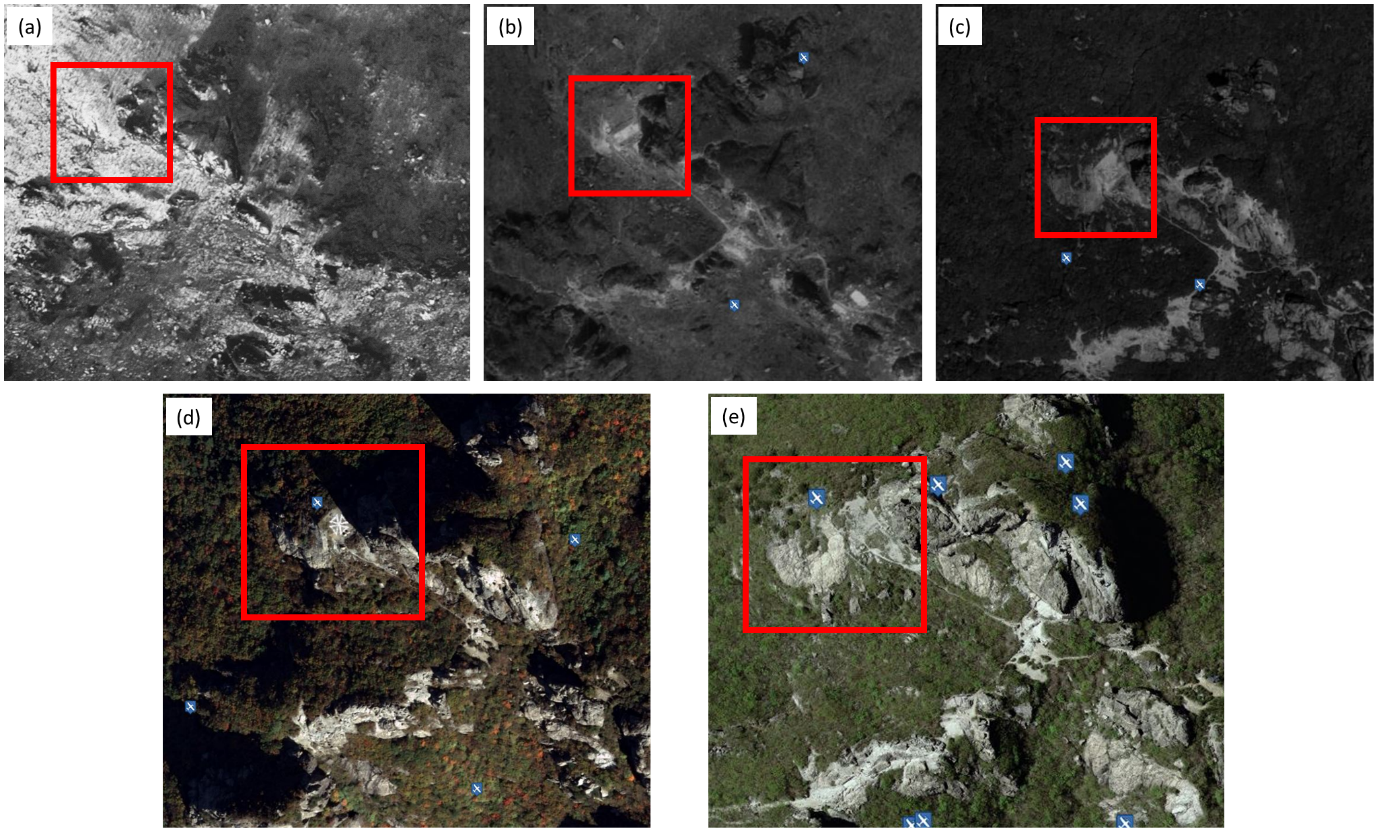


Figure S1. Historical changes in the study area (red square) (a: 1954; b: 1982; c: 1996; d: 2010; e: 2015). (Source: https://map.ngii.go.kr/ms/map/NlipMap.do, accessed on 8 April 2024).
